# Obesity-Related Indices Are Associated with Longitudinal Changes in Lung Function: A Large Taiwanese Population Follow-Up Study

**DOI:** 10.3390/nu13114055

**Published:** 2021-11-12

**Authors:** Yu-En Hsu, Szu-Chia Chen, Jiun-Hung Geng, Da-Wei Wu, Pei-Yu Wu, Jiun-Chi Huang

**Affiliations:** 1 Department of Post Baccalaureate Medicine, Kaohsiung Medical University, Kaohsiung 807, Taiwan; afrwtLJ@gmail.com; 2 Department of Internal Medicine, Kaohsiung Municipal Siaogang Hospital, Kaohsiung Medical University, Kaohsiung 812, Taiwan; scarchenone@yahoo.com.tw (S.-C.C.); u8900030@yahoo.com.tw (D.-W.W.); wpuw17@gmail.com (P.-Y.W.); 3 Division of Nephrology, Department of Internal Medicine, Kaohsiung Medical University Hospital, Kaohsiung Medical University, Kaohsiung 807, Taiwan; 4 Faculty of Medicine, College of Medicine, Kaohsiung Medical University, Kaohsiung 807, Taiwan; 5 Research Center for Environmental Medicine, Kaohsiung Medical University, Kaohsiung 807, Taiwan; 6 Department of Urology, Kaohsiung Municipal Siaogang Hospital, Kaohsiung Medical University, Kaohsiung 812, Taiwan; u9001090@gmail.com; 7 Department of Urology, Kaohsiung Medical University Hospital, Kaohsiung Medical University, Kaohsiung 807, Taiwan; 8 Division of Pulmonary and Critical Care Medicine, Department of Internal Medicine, Kaohsiung Medical University Hospital, Kaohsiung Medical University, Kaohsiung 807, Taiwan

**Keywords:** obesity-related index, lung function, follow-up, Taiwan Biobank

## Abstract

The global pandemic of obesity and the increasing incidence of chronic respiratory diseases are growing health concerns. The association between obesity and pulmonary function is uncertain. Therefore, this study aimed to explore associations between changes in lung function and obesity-related indices in a large longitudinal study. A total of 9059 participants with no personal histories of asthma, smoking, bronchitis, or emphysema were enrolled from the Taiwan Biobank and followed for 4 years. Lung function was assessed using spirometry measurements including forced expiratory volume in 1 s (FEV1) and forced vital capacity (FVC). Changes in FEV1/FVC (∆FEV1/FVC) between baseline and follow-up were calculated. The following obesity-related indices were studied: lipid accumulation product (LAP), body roundness index (BRI), conicity index (CI), body adiposity index (BAI), abdominal volume index (AVI), body mass index (BMI), waist–hip ratio (WHR), and waist-to-height ratio (WHtR). In multivariable analysis, the subjects with high BMI (*p* < 0.001), WHR (*p* < 0.001), WHtR (*p* < 0.001), LAP (*p* = 0.002), BRI (*p* < 0.001), CI (*p* = 0.005), BAI (*p* < 0.001), and AVI (*p* < 0.001) were significantly associated with a high baseline FEV1/FVC. After 4 years of follow-up, the subjects with high BMI (*p* < 0.001), WHR (*p* < 0.001), WHtR (*p* < 0.001), LAP (*p* = 0.001), BRI (*p* < 0.001), CI (*p* = 0.002), BAI (*p* < 0.001), and AVI (*p* < 0.001) were significantly associated with a low △FEV1/FVC. High obesity-related index values were associated with better baseline lung function and a rapid decrease in lung function at follow-up.

## 1. Introduction

The prevalence of chronic respiratory diseases has been increasing globally since 1990 [1]. Chronic obstructive pulmonary disease (COPD) is a common chronic respiratory disease and the leading cause of morbidity worldwide [1]. COPD is associated with an increased social burden and impaired quality of life, and it is an increasing problem due to the increasing global population and high smoking rates in Asia [2,3]. Complications associated with COPD include cor pulmonale [4], recurrent pneumonia [5], depression [6], lung cancer [7], respiratory failure [8], pneumothorax [9], anemia [10], and polycythemia [11]. Asthma is also a chronic respiratory disease that can lead to status asthmaticus or even be fatal if inadequately treated or controlled [12]. The known risk factors for chronic respiratory diseases include smoking, second-hand smoke, particulate matter, household air pollution, and occupational risks [13]. In patients with COPD, a rapid decline in forced expiratory volume in 1 s (FEV1) has been independently associated with more hospital admissions and mortality [14]. A study of young cystic fibrosis patients reported that female sex, increased cough frequency, productive cough, body mass index (BMI) < 66th percentile, and FEV1 ≥ 115% predicted were associated with a decline in FEV1 [15].

Obesity has also been associated with asthma through mechanical changes in the respiratory system. Such changes include limitations on displacement between the inspiration and expiratory phases of the diaphragm, leading to insufficient lung expansion and impaired airway dilation. This weakened dilation may lead to greater contraction of the smooth muscles of the airway, which can then increase airway responsiveness [16]. Anthropometric indices have been strongly correlated with metabolic syndrome, atherosclerotic cardiovascular diseases, and diabetes [17,18,19], including lipid accumulation product (LAP), body roundness index (BRI), conicity index (CI), body adiposity index (BAI), abdominal volume index (AVI), body mass index (BMI), waist–hip ratio (WHR), and waist-to-height ratio (WHtR). These indices can be quantified and calculated using waist circumference (WC), hip circumference (HC), body height (BH), body weight (BW), and levels of triglycerides (TGs) and high-density lipoprotein cholesterol (HDL-C) [17]. No previous large cohort follow-up investigations have explored the association among changes in lung function and obesity-related indices. Therefore, the aim of this study was to explore this association in a large longitudinal cohort of participants from the Taiwan Biobank (TWB).

## 2. Materials and Methods

### 2.1. TWB

The TWB is the largest biobank supported by the Taiwanese government. The TWB was established with the aim of recording lifestyle and genomic data on Taiwanese residents [20,21]. The TWB includes data on volunteers living in the community with an age between 30 and 70 years without a history of cancer. Each participant in the TWB provides written informed consent, after which in-person interviews and physical examinations are conducted and blood samples obtained.

During the physical examination, WC, HC, BH, and BW are measured. During the in-person interview, each participant completes a questionnaire that collects data on diet, lifestyle factors, family and personal medical histories, and personal information. In this study, regular exercise was defined as performing at least 30 min of physical activity thrice weekly. In addition, physical activity was defined as participating in activities such as yoga, cycling, playing a sport, computer-based dancing/exercise games/activities, hiking, swimming, and jogging. However, heavy manual labor or physical activity related to work was not recorded.

### 2.2. Study Participants

**Figure 1:**
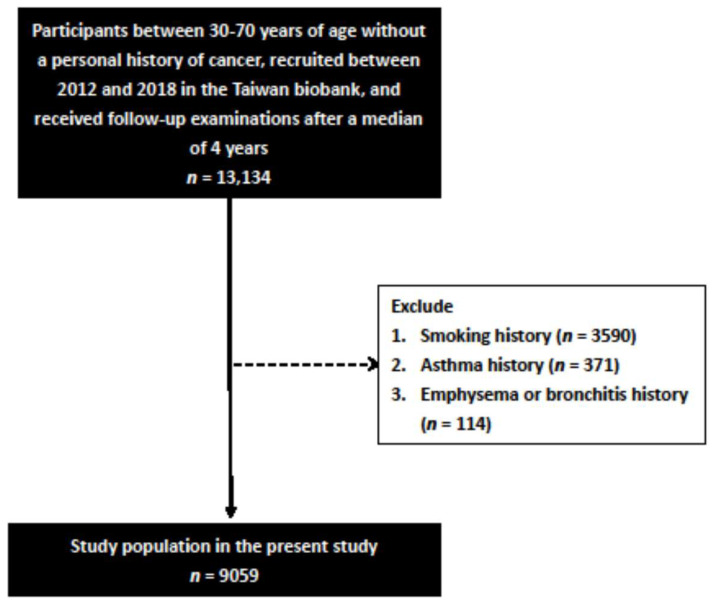
Flowchart of study population.

In this study, 27,033 participants with complete lung function data were included. Of these participants, 13,134 received follow-up examinations after a median of 4 years. The attrition rate was 51.4%. The 13,134 participants were aged between 30 and 70 years, and they all had complete follow-up spirometry data. We excluded those with a history of bronchitis or emphysema (*n* = 114), asthma (*n* = 371), and smoking (*n* = 3590), and enrolled the remaining 9059 participants (Figure 1).

### 2.3. Ethics Statement

The Ethics and Governance Council of the TWB and the Institutional Review Board (IRB) on Biomedical Science Research, Academia Sinica, Taiwan gave ethical approval for the TWB. In addition, the IRB of Kaohsiung Medical University Hospital approved this study (KMUHIRB-E(I)-20190398), which was conducted according to the Declaration of Helsinki.

### 2.4. Demographic, Medical, and Laboratory Data

Baseline data included sex, age, histories of diabetes mellitus, smoking, hypertension, asthma and emphysema or bronchitis, levels of total cholesterol, low-density lipoprotein cholesterol (LDL-C), HDL-C, TGs, hemoglobin, fasting glucose, uric acid and estimated glomerular filtration rate (eGFR), and systolic (SBP) and diastolic blood pressure (DBP). eGFR was calculated using the Modification of Diet in Renal Disease 4-variable equation [22].

### 2.5. Calculation of Obesity-Related Indices

The obesity-related indices were calculated as follows:

1. BMI = BW (kg)/BH^2^ (m).

2. WHR = WC (cm)/HC (cm).

3. WHtR = WC (cm)/BH (cm).

4. LAP = $\left({\mathrm{WC}}_{\left(\mathrm{cm}\right)}-65\right)\times {\mathrm{TG}}_{\left(\mathrm{mmol}/L\right)}$ in males, and LAP = $\left({\mathrm{WC}}_{(\mathrm{cm})}-58\right)\times {\mathrm{TG}}_{\mathrm{mmol}/L}$ in females [23].

5. BRI = $364.2-365.5\times \sqrt{1-{\left(\frac{\frac{{\mathrm{WC}}_{\left(m\right)}}{2\pi }}{0.5\times {\mathrm{BH}}_{\left(m\right)}}\right)}^{2}}$ [24].

6. CI was calculated using the Valdez equation [25]:

$$\mathrm{CI}\;=\;\frac{{\mathrm{WC}}_{\left(m\right)}}{0.109\times \sqrt{\frac{{\mathrm{BW}}_{\left(\mathrm{kg}\right)}}{{\mathrm{BH}}_{\left(m\right)}}}}$$

7. BAI was calculated according to the method of Bergman and colleagues as [26]:

$$\mathrm{BAI}\;=\;\frac{{\mathrm{HC}}_{\left(\mathrm{cm}\right)}}{{\mathrm{BH}}_{\left(m\right)}{}^{3/2}}-18$$

8. AVI = $\frac{2\times {\left(WC_{\left(\mathrm{cm}\right)}\right)}^{2}+0.7\times {\left(WC_{\left(\mathrm{cm}\right)}-{\mathrm{HC}}_{\left(\mathrm{cm}\right)}\right)}^{2}}{1000}$ [27].

### 2.6. Spirometry Measurements

All measurements of forced vital capacity (FVC) and FEV1 were made using a spirometer (Micro Labs Spirometer, Micro Medical Ltd., Rochester, Kent, UK) according to the American Thoracic Society and European Respiratory Society guidelines (2005) (FVC and FEV1 values within 100 mL or 5%) [28]. The best of 3 measurements was included in our analysis. Sex-, height-, and age-adjusted reference values based on Asian subjects were obtained and used to calculate predicted FEV1, percent predicted FEV1, predicted FVC, and percent predicted FVC with Spida 5 software (Micro Medical Ltd., Rochester, Kent, UK). Changes in FEV1/FVC were determined as the value at follow-up minus that at baseline.

### 2.7. Statistical Analysis

SPSS version 20 for Windows (SPSS Inc., Chicago, IL, USA) was used for all statistical analyses. Data are presented as percentages or means ± standard deviations. The chi-squared and independent *t* test were used to analyze between-group differences in categorical and continuous variables, respectively. Linear regression analysis was used to identify associations between variables and ∆FEV1/FVC. Significant variables in univariable analysis and each obesity-related index were entered into multivariable analysis. *p* values of < 0.05 were considered to be statistically significant.

## 3. Results

A total of 9059 participants (7245 females; 1814 males; mean age 51.0 ± 10.2 years) were enrolled and stratified into normal lung function (*n* = 6016 (66.4%), FEV1/FVC ≥ 70%, and FVC predicted ≥ 80%) and obstructive lung function (*n* = 3043 (33.6%), FEV1/FVC < 70%) groups.

### 3.1. Comparisons of Clinical Characteristics between the Normal and Obstructive Lung Function Groups

The obstructive lung function group was more predominantly female and had higher SBP and DBP and lower BW, WC, HC, hemoglobin, and uric acid than the normal lung function group (Table 1). In addition, the obstructive lung function group had lower values of FVC, FEV1, and FEV1/FVC at both baseline and follow-up. With regard to the obesity-related indices, the obstructive lung function group had lower AVI, CI, BRI, LAP, WHtR, WHR, and BMI than the normal lung function group.

### 3.2. Associations among the Obesity-Related Indices with Baseline FEV1/FVC

Univariable linear regression analysis showed that young age, male sex, low SBP, low DBP, and high uric acid were associated with a high baseline FEV1/FVC (Table 2). Multivariable linear regression analysis was performed with adjustments for age, sex, SBP, DBP, and uric acid to investigate associations among baseline FEV1/FVC and the obesity-related indices (Table 3). The results showed that the participants with high BMI (per 1 kg/m^2^; coefficient β, 0.303; *p* < 0.001), high WHR (per 1%; β, 0.123; *p* < 0.001), high WHtR (per 1%; β, 0.190; *p* < 0.001), high LAP (per 10; β, 0.245; *p* = 0.002), high BRI (per 1; β, 0.565; *p* < 0.001), high CI (per 0.1; β, 0.694; *p* = 0.005), high BAI (per 1; β, 0.263; *p* < 0.001), and high AVI (per 1; β, 0.296; *p* < 0.001) were significantly associated with a high FEV1/FVC.

We further performed subgroup analysis in the two lung function groups (Supplementary Table S1). We found that after multivariable analysis, high BMI, high WHR, high WHtR, high LAP, high BRI, high CI, high BAI, and high AVI were significantly associated with a high FEV1/FVC in the normal lung function group. However, no significant associations were noted between any obesity index and FEV1/FVC in the obstructive lung function group.

We further performed subgroup analysis by gender (Supplementary Table S3). Multivariable analysis showed that high levels of the obesity indices were significantly associated with a high FEV1/FVC in both the male and female participants; however, there was no significant association between CI and FEV1/FVC in the female participants.

We further performed subgroup analysis in different age groups (Supplementary Table S5). Multivariable analysis showed that high levels of the obesity indices were significantly associated with a high FEV1/FVC in those aged ≥ or <52 years (median); however, there were no significant associations between WHR, LAP, and CI and FEV1/FVC in those aged ≥52 years.

### 3.3. Associations among the Obesity-Related Indices with ∆FEV1/FVC

Univariable linear regression analysis showed that male sex, low SBP, low DBP, low total cholesterol, and low eGFR were associated with a low △FEV1/FVC (Table 4). Multivariable linear regression analysis was performed with adjustments for age, sex, SBP, DBP, total cholesterol, and eGFR (Table 5). The results showed that high BMI (per 1 kg/m^2^; β, −0.280; *p* < 0.001), high WHR (per 1%; β, −0.123; *p* < 0.001), high WHtR (per 1%; β, −0.190; *p* < 0.001), high LAP (per 10; β, −0.284; *p* = 0.001), high BRI (per 1; β, −0.567; *p* < 0.001), high CI (per 0.1; β, −0.855; *p* = 0.002), high BAI (per 1; β, −0.272; *p* < 0.001), and high AVI (per 1; β, −0.306; *p* < 0.001) were significantly associated with a low △FEV1/FVC.

We further performed subgroup analysis in the two lung function groups (Supplementary Table S2). We found that after multivariable analysis, high WHR, high WHtR, and high CI were significantly associated with a low ∆FEV1/FVC in the normal lung function group. However, no significant associations were noted between any obesity index and ∆FEV1/FVC in the obstructive lung function group.

We further performed subgroup analysis by gender (Supplementary Table S4). Multivariable analysis showed that high levels of the obesity indices were significantly associated with a low ∆FEV1/FVC in both the male and female participants; however, there was no significant association between LAP and BAI with ∆FEV1/FVC in the male participants.

We further performed subgroup analysis by different age groups (Supplementary Table S6). Multivariable analysis showed that high levels of the obesity indices were significantly associated with a low ∆FEV1/FVC in those aged ≥ or <52 years (median); however, there was no significant association between WHR and ∆FEV1/FVC in those aged ≥52 years.

## 4. Discussion

In this study, we investigated the associations among various obesity-related indices and changes in FEV1/FVC after 4 years of follow-up. We found that high values of all the studied obesity-related indices (AVI, BAI, CI, BRI, LAP, WHtR, WHR, and BMI) were associated with a high baseline FEV1/FVC and low △FEV1/FVC.

With regard to the associations between high levels of the obesity-related indices and a high baseline FEV1/FVC, a previous study of 190 patients with stable COPD reported that overweight/obese patients (BMI ≥ 25 kg/m^2^) had better lung function, as reflected by a higher baseline FEV1, than normal-weight patients [29]. In addition, the overweight/obese patients had a better peak work rate, which is a marker of peak exercise capacity and was defined as maximum exercise workload that could be tolerated for at least 30 s. Moreover, a recent study on medical students in Pakistan reported a positive correlation between WHR and peak expiratory flow rate in those with a normal BMI [30]. Furthermore, Haider et al. found a positive association between BMI and FEV1/FVC ratio, and also similar non-linear associations with anthropometric indices including BRI and WC [31]. These results may be explained by an increase in FEV1 or decrease in FVC. First, if a high baseline FEV1/FVC ratio is caused by an increase in FEV1, it is important to consider body fat. The trends of obesity-related indices such as AVI, BAI, CI, BRI, LAP, WHtR, and WHR often follow that of BMI. A large BMI means that there is a large amount of fat and muscle tissue [32], and patients with COPD and a greater amount of muscle tissue have been associated with better survival [29]. Furthermore, Cornell et al. reported that an adequate amount of fat (defined as a BMI < 30 kg/m^2^ [33]) can help the body by providing energy to produce less CO_2_ [34], which is essential for COPD patients because of their impaired ability to exhale CO_2_ [34]. Second, if a high baseline FEV1/FVC ratio is caused by a low FVC, it may be due to the mechanical and inflammatory effects of fat tissue on lung function. A possible mechanism is that when fat accumulates in the mediastinum and abdominal and thoracic cavities, it causes an elevation of the diaphragm that limits its downward expansion, leading to increased pleural pressure and decreased functional residual capacity [35]. Another possible mechanism is insulin resistance, in which excessive fat accumulates in the body [36]. The Normative Aging Study in nondiabetic participants [37] and Strong Heart Study in Adult American Indians [38] demonstrated that insulin resistance was negatively correlated with FEV1 and FVC. Moreover, studies of healthy participants such as Korean adults without type 2 diabetes have reported that insulin resistance more profoundly affects FVC [39,40] and people with asthma [41], leading to a high FEV1/FVC ratio. Low-grade chronic inflammation, which occurs in obese people without overt infection, may be the underlying mechanism. A third possible mechanism is that in addition to the association between obesity and low FEV1, low FVC, and high FEV1/FVC ratio, it may also be associated with a higher level of C-reactive protein (CRP) [31]. A cross-sectional study including individuals with chronic spinal cord injuries also reported an association between a high CRP level and low FVC [42]. Taken together, these possible mechanisms may partly explain the associations among high obesity-related index values and high baseline FEV1/FVC in this study.

Another important finding of this study is that high values of the obesity-related indices were associated with a rapid decrease in FEV1/FVC. A longitudinal study of Caucasian males with a mean age of 49 years and chronic spinal cord injuries reported that an increase in BMI was a predictor of decline in FEV1/FVC [43]. In addition, a recent study of 3442 participants in Wuhan-Zhuhai reported that 1% increases in WHtR and WHR were associated with 14.23 mL and 5.42 mL decreases in FEV1, and 16.92 mL and 5.70 mL decreases in FVC [44], respectively, leading to a decrease in FEV1/FVC. Moreover, a study of 1276 asthmatic adults from the National Health and Nutrition Examination Survey 2009–2012 database also revealed significant negative correlations between BMI, WC, and WHtR and FEV1 [45]. Furthermore, the Effectiveness of Smoking Cessation Advice Combined With Spirometric Results in Adult Smokers randomized controlled trial study in Spain, which included 738 smokers without respiratory diseases and aged 35 to 70 years, reported an inverse association between FEV1% and BMI, WC, and WHtR in the males, and an inverse association between FEV1% and BMI and WC in the females [46]. One possible mechanism for these results may be that the BMI of obese and overweight patients tends to increase over time [16], along with other obesity-related indices such as WC and WHtR. The relatively mild inflammatory reactions caused by fat tissue may then worsen and affect the originally better lung function. Even in patients who do not gain much weight over time, the large initial degree of inflammation may take a toll on lung function in subsequent years. Another possible mechanism is that adipose tissue accumulation may lead to impaired lung function [47]. Fat accumulation in the diaphragm and chest wall limits displacement between the inspiration and expiratory phases of the diaphragm, thereby decreasing thoracic compliance [48]. A decrease in lung volume or impaired thoracic compliance or both may then result in muscle inefficiency, leading to weakened respiratory muscles [49], which may be reflected in a decrease in FEV1 [50]. Another possible mechanism may be an inflammation-mediated response. The English Longitudinal Study of Ageing demonstrated a negative correlation between CRP and FEV1 [51]. Adipose tissue accumulation may lead to metabolic dysfunction, such as increased serum levels of free fatty acids and their breakdown. An increase in triacylglycerol creates large adipocytes that can cause cellular stress responses, which may then activate inflammatory signaling pathways [52]. Subsequently, activated macrophages lead to an increased expression of CRP [52]. In addition, chronic inflammation can also lead to the senescence of alveolar endothelial cells [53], and impaired endothelial cells may lead to pulmonary vascular filtration and subsequently lung tissue damage [44].

We found similar results in that the obesity-related indices were associated with a high baseline FEV1/FVC and a rapid decrease in FEV1/FVC in the normal lung function group. However, no significant association was noted between obesity with FEV1/FVC and △FEV1/FVC in the obstructive lung function group. A multicenter study of 6610 subjects in three age strata reported a mean decline in FEV1 among the incident cases of 51 mL/year (mild COPD 43 mL/year, moderate COPD 54 mL/year, and severe COPD 102 mL/year) [54]. Furthermore, in a 2016 study of 617 participants, the mild COPD group (72 participants) showed an even higher mean decline in FEV1 (129 mL/year) [55]. In a 2021 cohort study of 1746 participants, each 1-cm increase in WC decreased FEV1 by 6 mL in men and by 2 mL in women [56]. In addition, in the Coronary Artery Risk Development in Young Adults (CARDIA) study, the mean decline in FEV1 among participants with a baseline BMI ≥ 26.4 kg/m^2^ was 6.4 mL/year [57]. We hypothesize that the effect of COPD on FEV1 and FVC is much greater than the effect of obesity on FEV1 and FVC, and the former may mask the latter. The impact of both obstructive lung disease and obesity should be taken into consideration when evaluating lung function.

The strengths of the present study include the large-scale investigation and follow-up data. However, there are also several limitations. First, the TWB does not include data on medications, and certain medications may affect changes in lung function. Second, approximately half of the participants in the TWB return for follow-up examinations, which may have resulted in sample bias and may have affected the interpretation of our results. In addition, as all participants in the TWB are of Chinese ethnicity, our findings may not be generalizable to other populations. Finally, the TWB includes data on community-based volunteers aged 30 to 70 years with no history of cancer. Volunteer bias could have occurred at any stage of the trial from recruitment to retention to follow-up. Differences between volunteers and the target population may not have been restricted to socio-demographic factors, and may also have included attitudes towards the trial and institutions involved. Volunteer bias may also be related to the diseases or conditions being studied. Therefore, volunteer bias may have affected the interpretation of our results.

In conclusion, we found that high values of obesity-related indices including AVI, BAI, CI, BRI, LAP, WHtR, WHR, and BMI were associated with better baseline lung function but a rapid decrease in lung function after follow-up. These findings highlight that: (1) central obesity may truly benefit baseline FEV1/FVC; and (2) the FEV1/FVC ratio may not truly reveal the underlying lung function, because an increased FEV1/FVC may be caused by a decrease in FVC. Our results showed that good baseline pulmonary function was associated with not being overweight or underweight, and that this could prevent a decline in pulmonary function over time.

## Figures and Tables

**Table 1:** Comparison of clinical characteristics among participants according to lung function.

Characteristics	Normal (*n* = 6016)	Obstructive (*n* = 3043)	*p*
Age (year)	50.9 ± 10.1	51.2 ± 10.5	0.275
Male gender (%)	20.9	18.2	0.003
DM (%)	4.5	3.8	0.115
Hypertension (%)	11.2	11.3	0.867
Regular exercise habits (%)	49.7	48.8	0.406
SBP (mmHg)	115.6 ± 17.5	116.7 ± 17.9	0.005
DBP (mmHg)	71.0 ± 10.6	71.6 ± 10.6	0.007
Body height (cm)	159.5 ± 7.5	159.3 ± 7.3	0.220
Body weight (kg)	60.6 ± 11.0	59.6 ± 10.5	<0.001
Waist circumference (cm)	81.9 ± 9.5	81.1 ± 9.3	<0.001
Hip circumference (cm)	95.3 ± 6.7	94.9 ± 6.6	0.007
Laboratory parameters			
Fasting glucose (mg/dL)	94.9 ± 17.7	94.3 ± 17.3	0.139
Hemoglobin (g/dL)	13.4 ± 1.5	13.4 ± 1.4	0.037
Triglyceride (mg/dL)	106.8 ± 74.5	104.2 ± 69.1	0.105
Total cholesterol (mg/dL)	196.4 ± 35.3	195.9 ± 34.9	0.568
HDL-cholesterol (mg/dL)	56.1 ± 13.1	56.1 ± 13.0	0.853
LDL-cholesterol (mg/dL)	121.7 ± 31.3	121.0 ± 30.9	0.560
eGFR (mL/min/1.73 m^2^)	110.7 ± 25.5	111.5 ± 24.7	0.153
Uric acid (mg/dL)	5.24 ± 1.31	5.16 ± 1.30	0.006
Lung function			
FVC (L, baseline)	2.68 ± 0.71	2.58 ± 0.73	<0.001
FVC (L, follow-up)	2.48 ± 0.69	2.44 ± 0.70	0.003
FEV1 (L, baseline)	2.25 ± 0.61	1.31 ± 0.53	<0.001
FEV1 (L, follow-up)	2.17 ± 0.63	2.10 ± 0.66	<0.001
FEV1/FVC (%, baseline)	84.02 ± 6.21	50.55 ± 13.65	<0.001
FEV1/FVC (%, follow-up)	87.86 ± 9.63	86.31 ± 12.18	<0.001
Obesity-related indices			
BMI (kg/m^2^)	23.8 ± 3.5	23.4 ± 3.4	<0.001
WHR (%)	85.8 ± 6.7	85.3 ± 6.8	0.001
WHtR (%)	51.4 ± 6.0	51.0 ± 5.9	0.001
LAP	29.0 ± 27.5	27.6 ± 25.4	0.022
BRI	6.6 ± 1.8	6.4 ± 1.8	<0.001
CI	1.222 ± 0.082	1.219 ± 0.084	0.037
BAI	29.4 ± 4.0	29.3 ± 3.9	0.185
AVI	13.7 ± 3.2	13.5 ± 3.1	<0.001

Abbreviations. DM, diabetes mellitus; SBP, systolic blood pressure; DBP, diastolic blood pressure; HDL, high-density lipoprotein; LDL, low-density lipoprotein; eGFR, estimated glomerular filtration rate; FVC, forced vital capacity, FEV1, forced expiratory volume in 1 s; BMI, body mass index; WHR, waist–hip ratio; WHtR, waist-to-height ratio; LAP, lipid accumulation product; BRI, body roundness index; CI, conicity index; BAI, body adiposity index; AVI, abdominal volume index. Normal lung function was defined as FEV1/FVC ≥ 70% and FVC–predict ≥ 80%; obstructive lung function was defined as FEV1/FVC < 70%.

**Table 2:** Determinants for baseline FEV1/FVC using univariable linear regression analysis.

Characteristics	Univariable	
	Unstandardized Coefficient β (95% CI)	*p*
Age (per 1 year)	−0.053 (−0.090, −0.016)	0.005
Male (vs. female)	1.154 (0.208, 2.100)	0.017
DM	1.174 (−0.697, 3.044)	0.219
Hypertension	−0.341 (−1.539, 0.857)	0.577
Regular exercise habits	−0.078 (−0.835, 0.680)	0.841
SBP (per 1 mmHg)	−0.044 (−0.066, −0.023)	<0.001
DBP (per 1 mmHg)	−0.063 (−0.099, −0.028)	0.001
Laboratory parameters		
Fasting glucose (per 1 mg/dL)	0.013 (−0.008, 0.035)	0.230
Hemoglobin (per 1 g/dL)	0.227 (−0.033, 0.488)	0.088
Triglyceride (per 1 mg/dL)	0.005 (0, 0.010)	0.069
Total cholesterol (per 1 mg/dL)	−0.004 (−0.014, 0.007)	0.510
HDL-cholesterol (per 1 mg/dL)	−0.006 (−0.035, 0.023)	0.675
LDL-cholesterol (per 1 mg/dL)	7.781 × 10^−5^ (−0.012, 0.012)	0.990
eGFR (per 1 mL/min/1.73 m^2^)	−0.001 (−0.016, 0.014)	0.854
Uric acid (per 1 mg/dL)	0.396 (0.106, 0.686)	0.007

Values expressed as unstandardized coefficient β and 95% confidence interval (CI). Abbreviations are the same as in Table 1.

**Table 3:** Association of obesity-related indices with baseline FEV1/FVC using multivariable linear regression analysis.

Obesity-Related Indices	Multivariable		Adjusted R Square
	Unstandardized Coefficient β (95% CI)	*p*	
BMI (per 1 kg/m^2^)	0.303 (0.180, 0.426)	<0.001	0.6%
WHR (per 1%)	0.123 (0.059, 0.186)	<0.001	0.5%
WHtR (per 1%)	0.190 (0.120, 0.261)	<0.001	0.7%
LAP (per 1)	0.245 (0.092, 0.397)	0.002	0.5%
BRI (per 1)	0.565 (0.336, 0.793)	<0.001	0.6%
CI (per 0.1)	0.694 (0.210, 1.178)	0.005	0.4%
BAI (per 1)	0.263 (0.152, 0.374)	<0.001	0.6%
AVI (per 1)	0.296 (0.161, 0.431)	<0.001	0.6%

Values expressed as unstandardized coefficient β and 95% confidence interval (CI). Abbreviations are the same as in Table 1. Multivariable model: adjusted for age, sex, SBP, DBP, and uric acid (significant variables of Table 2).

**Table 4:** Determinants for ∆FEV1/FVC using univariable linear regression analysis.

Characteristics	Univariable	
	Unstandardized Coefficient β (95% CI)	*p*
Age (per 1 year)	0.020 (−0.020, 0.061)	0.325
Male (vs. female)	−1.717 (−2.761, −0.674)	0.001
DM	−0.707 (−2.771, 1.357)	0.502
Hypertension	0.347 (−0.975, 1.669)	0.607
Regular exercise habits	−0.207 (−1.043, 0.629)	0.627
SBP (per 1 mmHg)	0.073 (0.050, 0.097)	<0.001
DBP (per 1 mmHg)	0.113 (0.073, 0.152)	<0.001
Laboratory parameters		
Fasting glucose (per 1 mg/dL)	−0.012 (−0.036, 0.012)	0.317
Hemoglobin (per 1 g/dL)	−0.246 (−0.534, 0.042)	0.094
Triglyceride (per 1 mg/dL)	−0.004 (−0.010, 0.002)	0.169
Total cholesterol (per 1 mg/dL)	0.012 (0, 0.024)	0.045
HDL-cholesterol (per 1 mg/dL)	−0.021 (−0.053, 0.011)	0.202
LDL-cholesterol (per 1 mg/dL)	0.006 (−0.008, 0.019)	0.401
eGFR (per 1 mL/min/1.73 m^2^)	0.034 (0.018, 0.051)	<0.001
Uric acid (per 1 mg/dL)	−0.248 (−0.568, 0.072)	0.128

Values expressed as unstandardized coefficient β and 95% confidence interval (CI). Abbreviations are the same as in Table 1.

**Table 5:** Association of obesity-related indices with ∆FEV1/FVC using multivariable linear regression analysis.

Obesity-Related Indices	Multivariable		Adjusted R Square
	Unstandardized Coefficient β (95% CI)	*p*	
BMI (per 1 kg/m^2^)	−0.280 (−0.409, −0.151)	<0.001	1.0%
WHR (per 1%)	−0.123 (−0.192, −0.055)	<0.001	0.8%
WHtR (per 1%)	−0.190 (−0.265, −0.115)	<0.001	1.1%
LAP (per 1)	−0.284 (−0.448, −0.120)	0.001	0.9%
BRI (per 1)	−0.567 (−0.808, −0.325)	<0.001	1.0%
CI (per 0.1)	−0.855 (−1.385, −0.326)	0.002	0.9%
BAI (per 1)	−0.272 (−0.392, −0.153)	<0.001	1.0%
AVI (per 1)	−0.306 (−0.449, −0.163)	<0.001	1.0%

Values expressed as unstandardized coefficient β and 95% confidence interval (CI). Abbreviations are the same as in Table 1. Multivariable model: adjusted for age, sex, SBP, DBP, total cholesterol, and eGFR (significant variables of Table 4).

## Data Availability

The data underlying this study is from the Taiwan Biobank. Due to restrictions placed on the data by the Personal Information Protection Act of Taiwan, the minimal data set cannot be made publicly available. Data may be available upon request to interested researchers. Please send data requests to: Szu-Chia Chen, PhD, MD. Division of Nephrology, Department of Internal Medicine, Kaohsiung Medical University Hospital, Kaohsiung Medical University.

## Supplementary Materials

The following are available online at https://www.mdpi.com/article/10.3390/nu13114055/s1, Table S1: Determinants for FEV1/FVC in different lung function group using multivariable linear regression analysis, Table S2: Determinants for △FEV1/FVC in different lung function group using multivariable linear regression analysis, Table S3: Determinants for FEV1/FVC in different gender group using multivariable linear regression analysis, Table S4: Determinants for △FEV1/FVC in different gender using multivariable linear regression analysis, Table S5: Determinants for FEV1/FVC in different age group using multivariable linear regression analysis, Table S6: Determinants for △FEV1/FVC in different age group using multivariable linear regression analysis.
