# Supplementary material for: Obesity-Related Indices Are Associated with Longitudinal Changes in Lung Function: A Large Taiwanese Population Follow-Up Study
Source: Nutrients. 2021 Nov 12;13(11):4055. doi: 10.3390/nu13114055 (PMC8624262; doi:10.3390/nu13114055)
Supplement: Supplementary file 1 [file nutrients-13-04055-s001.zip › nutrients-1401910-supplementary.pdf]

Supplementary Table S1. Determinants for FEV1/FVC in different lung function group using multivariable linear regression analysis

Characteristics	Normal (n = 6016)		Obstructive (n = 3043)	
	Multivariable		Multivariable	
	Unstandardized coefficient β (95% CI)	*p*	Unstandardized coefficient β (95% CI)	*p*
BMI (per 1 kg/m²)	0.084 (0.034, 0.135)	0.001	0.029 (-0.133, 0.190)	0.728
WHR (per 1%)	0.052 (0.026, 0.079)	< 0.001	-0.032 (-0.113, 0.048)	0.434
WHtR (per 1%)	0.070 (0.041, 0.099)	< 0.001	0.014 (-0.078, 0.105)	0.768
LAP (per 1)	0.008 (0.001, 0.014)	0.015	0.004 (-0.017, 0.025)	0.713
BRI (per 1)	0.186 (0.092, 0.280)	< 0.001	0.042 (-0.257, 0.340)	0.874
CI (per 0.1)	0.267 (0.066, 0.469)	0.009	-0.030 (-0.645, 0.585)	0.925
BAI (per 1)	0.102 (0.056, 0.148)	< 0.001	0.097 (-0.046, 0.241)	0.184
AVI (per 1)	0.079 (0.023, 0.134)	0.005	0.017 (-0.160, 0.194)	0.849

Values expressed as unstandardized coefficient β and 95% confidence interval (CI).

Multivariable model: adjusted for age, sex, SBP, DBP and uric acid.

Supplementary Table S2. Determinants for △FEV1/FVC in different lung function group using multivariable linear regression analysis

Characteristics	Normal (n = 6016)		Obstructive (n = 3043)	
	Multivariable		Multivariable	
	Unstandardized coefficient β (95% CI)	*p*	Unstandardized coefficient β (95% CI)	*p*
BMI (per 1 kg/m^2)	-0.029 (-0.012, 0.053)	0.486	-0.083 (00.285, 0.119)	0.420
WHR (per 1%)	-0.049 (-0.093, -0.004)	0.031	0.038 (-0.066, 0.142)	0.473
WHtR (per 1%)	-0.055 (-0.103, -0.007)	0.024	-0.041 (-0.156, 0.075)	0.493
LAP (per 1)	-0.006 (-0.017, 0.004)	0.230	-0.016 (-0.043, 0.010)	0.235
BRI (per 1)	-0.142 (-0.297, 0.012)	0.071	-0.124 (-0.501, 0.253)	0.518
CI (per 0.1)	-0.425 (-0.768, -0.082)	0.015	-0.006 (-0.809, 0.797)	0.988
BAI (per 1)	-0.072 (-0.149, 0.004)	0.064	-0.172 (-0.357, 0.013)	0.068
AVI (per 1)	-0.065 (-0.157, 0.026)	0.160	-0.063 (-0.286, 0.160)	0.580

Values expressed as unstandardized coefficient β and 95% confidence interval (CI).

Multivariable model: adjusted for age, sex, SBP, DBP, total cholesterol and eGFR.

Supplementary Table S3. Determinants for FEV1/FVC in different gender group using multivariable linear regression analysis

Characteristics	Male (n = 1814)		Female (n = 7245)	
	Multivariable		Multivariable	
	Unstandardized coefficient β (95% CI)	*p*	Unstandardized coefficient β (95% CI)	*p*
BMI (per 1 kg/m^2)	0.423 (0.140, 0.705)	0.003	0.271 (0.134, 0.408)	< 0.001
WHR (per 1%)	0.254 (0.085, 0.424)	0.003	0.100 (0.032, 0.169)	0.004
WHtR (per 1%)	0.325 (0.145, 0.505)	< 0.001	0.164 (0.087, 0.241)	< 0.001
LAP (per 1)	0.041 (0.010, 0.072)	0.010	0.019 (0.001, 0.037)	0.033
BRI (per 1)	0.988 (0.438, 1.538)	< 0.001	0.472 (0.220, 0.723)	< 0.001
CI (per 0.1)	2.004 (0.603, 3.405)	0.005	0.512 (-0.005, 1.029)	0.052
BAI (per 1)	0.362 (0.073, 0.651)	0.014	0.242 (0.121, 0.362)	< 0.001
AVI (per 1)	0.505 (0.208, 0.803)	0.001	0.240 (0.088, 0.392)	0.002

Values expressed as unstandardized coefficient β and 95% confidence interval (CI).

Multivariable model: adjusted for age, sex, SBP, DBP and uric acid.

Supplementary Table S4. Determinants for △FEV1/FVC in different gender using multivariable linear regression analysis

Characteristics	Male (n = 1814)		Female (n = 7245)	
	Multivariable		Multivariable	
	Unstandardized coefficient β (95% CI)	*p*	Unstandardized coefficient β (95% CI)	*p*
BMI (per 1 kg/m²)	-0.323 (-0.623, -0.025)	0.034	-0.273 (-0.416, -0.130)	< 0.001
WHR (per 1%)	-0.274 (-0.456, -0.093)	0.003	-0.102 (-0.176, -0.027)	0.007
WHtR (per 1%)	-0.290 (-0.481, -0.100)	0.003	-0.174 (-0.256, -0.092)	< 0.001
LAP (per 1)	-0.031 (-0.065, 0.003)	0.071	-0.028 (-0.047, -0.009)	0.004
BRI (per 1)	-0.900 (-1.483, -0.316)	0.003	-0.507 (-0.774. -0.241)	< 0.001
CI (per 0.1)	-2.389 (-3.898, -0.881)	0.002	-0.658 (-1.225, -0.090)	0.023
BAI (per 1)	-0.252 (-0.561, 0.057)	0.110	-0.273 (-0.403, -0.143)	< 0.001
AVI (per 1)	-0.480 (-0.796, -0.164)	0.003	-0.268 (-0.429, -0.108)	0.001

Values expressed as unstandardized coefficient β and 95% confidence interval (CI).

Multivariable model: adjusted for age, sex, SBP, DBP, total cholesterol and eGFR.

Supplementary Table S5. Determinants for FEV1/FVC in different age group using multivariable linear regression analysis

Characteristics	Age ≥ 52 years (n = 4801)		Age < 52 years (n = 4258)	
	Multivariable		Multivariable	
	Unstandardized coefficient β (95% CI)	*p*	Unstandardized coefficient β (95% CI)	*p*
BMI (per 1 kg/m²)	0.352 (0.177, 0.527)	< 0.001	0.239 (0.065, 0.413)	0.007
WHR (per 1%)	0.064 (-0.021, 0.150)	0.138	0.203 (0.108, 0.298)	< 0.001
WHtR (per 1%)	0.190 (0.094, 0.286)	< 0.001	0.199 (0.095, 0.303)	< 0.001
LAP (per 1)	0.018 (-0.002, 0.038)	0.075	0.032 (0.008, 0.057)	0.009
BRI (per 1)	0.567 (0.257, 0.876)	< 0.001	0.595 (0.255, 0.934)	00.001
CI (per 0.1)	0.575 (0.068, 1.219)	0.080	1.013 (0.274, 1.753)	0.007
BAI (per 1)	0.355 (0.201, 0.508)	< 0.001	0.166 (0.005, 0.327)	0.043
AVI (per 1)	0.315 (0.129, 0.500)	0.001	0.292 (0.094, 0.490)	0.004

Values expressed as unstandardized coefficient β and 95% confidence interval (CI).

Multivariable model: adjusted for age, sex, SBP, DBP and uric acid.

Supplementary Table S6. Determinants for △FEV1/FVC in different age group using multivariable linear regression analysis

Characteristics	Age ≥ 52 years (n = 4801)		Age < 52 years (n = 4258)	
	Multivariable		Multivariable	
	Unstandardized coefficient β (95% CI)	*p*	Unstandardized coefficient β (95% CI)	*p*
BMI (per 1 kg/m²)	-0.315 (-0.495, 0.135)	0.001	-0.256 (-0.444, -0.069)	0.007
WHR (per 1%)	-0.070 (-0.160, 0.021)	0.130	-0.208 (-0.314, -0.103)	< 0.001
WHtR (per 1%)	-0.192 (-0.292, -0.092)	< 0.001	-0.207 (-0.321, -0.094)	< 0.001
LAP (per 1)	-0.027 (-0.048, -0.006)	0.011	-0.033 (-0.060, -0.007)	0.014
BRI (per 1)	-0.582 (-0.903, -0.260)	< 0.001	-0.615 (-0.984, -0.247)	0.001
CI (per 0.1)	-0.794 (-1.485, -0.103)	0.024	-1.115 (-1.943, -0.287)	0.008
BAI (per 1)	-0.356 (-0.518, -0.194)	< 0.001	-0.193 (-0.370, -0.016)	0.033
AVI (per 1)	-0.337 (-0.530, -0.144)	0.001	-0.307 (-0.522, -0.093)	0.005

Values expressed as unstandardized coefficient β and 95% confidence interval (CI).

Multivariable model: adjusted for age, sex, SBP, DBP, total cholesterol and eGFR.
